# Social Advertising Effectiveness in Driving Action: A Study of Positive, Negative and Coactive Appeals on Social Media

**DOI:** 10.3390/ijerph18115954

**Published:** 2021-06-01

**Authors:** Murooj Yousef, Timo Dietrich, Sharyn Rundle-Thiele

**Affiliations:** Social Marketing @ Griffith, Nathan Campus, Griffith Business School, Griffith University, Brisbane, QLD 4111, Australia; t.dietrich@griffith.edu.au (T.D.); s.rundle-thiele@griffith.edu.au (S.R.-T.)

**Keywords:** emotional appeals, social advertising, behavior change, social media, advertising effectiveness, charity advertising, environmental advertising

## Abstract

Background: Social media offers a cost-effective and wide-reaching advertising platform for marketers. Objectively testing the effectiveness of social media advertising remains difficult due to a lack of guiding frameworks and applicable behavioral measures. This study examines advertising appeals’ effectiveness in driving engagement and actions on and beyond social media platforms. Method: In an experiment, positive, negative and coactive ads were shared on social media and promoted for a week. The three ads were controlled in an A/B testing experiment to ensure applicable comparison. Measures used included impressions, likes, shares and clicks following the multi-actor social media engagement framework. Data were extracted using Facebook ads manager and website data. Significance was tested through a series of chi-square tests. Results: The promoted ads reached over 21,000 users. Significant effect was found for appeal type on engagement and behavioral actions. The findings support the use of negative advertising appeals over positive and coactive appeals. Conclusion: Practically, in the charity and environment context, advertisers aiming to drive engagement on social media as well as behavioral actions beyond social media should consider negative advertising appeals. Theoretically, this study demonstrates the value of using the multi-actor social media engagement framework to test advertising appeal effectiveness. Further, this study proposes an extension to evaluate behavioral outcomes.

## 1. Introduction

The popularity of social media is growing with advertisers utilizing different platforms to drive online and offline customer engagement [1,2]. As the third largest advertising channel, social media accounts for 13% of global advertising spending [3]. In 2019, Australian brands spent AUD 2.4 billion on social media advertisements, making social media the second highest expenditure category in digital advertising spending after paid search [4]. Since the COVID-19 pandemic erupted across the world, social media played a crucial role in disseminating information. Research found social media to be the most rapid digital tool in spreading information regarding the virus, which helped reach and educate specific audiences, such as front-line workers [5]. With some drawbacks due to the publication of misinformative facts and knowledge, social media platforms remain a major communication platform for scientists, organizations and governments to reach different audience groups and create highly persuasive outcomes [6]. Similarly, advertisers invest in social media platforms, seeking attention, engagement and action in online and offline, making a clear understanding of social media advertising’s effectiveness of paramount importance.

It is established that emotional appeal messages perform better on social media than rational appeals. Evidence suggests emotional appeals are more likely to achieve engagement and virality [1,7]. However, less is known about the effectiveness of positive, negative and coactive emotional appeals, with the best approach to take remaining unresolved. Recent studies have explored positive versus negative appeals’ effectiveness, with mixed and inconsistent results [8,9]. In fact, examination of coactive appeals has been neglected in contrast to efforts directed at examining positive and negative appeals, further limiting practitioner guidance. This study aims to empirically test the effectiveness of three advertising appeals (i.e., positive, negative and coactive) delivered via social media. Effectiveness is evaluated using online engagement [10] and behavioral actions.

### Social Media Advertising

When creating social media ads, advertisers reportedly prioritize online engagement and utilize this measure to evaluate social media advertising success [11,12]. Quantitative online engagement metrics that social media platforms provide such as likes, comments, shares and clicks are key measures of online engagement. While such metrics provide insights into online engagement, they do not identify actual behaviors taken in response to viewed social media advertisements. To compensate for this omission, scholars have utilized separate data collection tools (i.e., surveys) to evaluate social media effectiveness, relying on consumer self-reports of actions taken following exposure to an advertisement [1]. The relationship between online engagement (e.g., likes, comments and shares) and behavior (e.g., donations) has received less attention. Therefore, there is limited understanding of social media advertising’s effect on actual behavior. Further, the relationship between online engagement and behavior is not reflected in social media marketing models [10] and models derived from empirical-based evaluation are needed to address this gap. To this end, the current study investigates social media advertising appeals, comparing three appeals’ (negative, positive and coactive) effects on online engagement and prosocial behavior. Drawing on empirical results, this paper proposes an extension to the social media multi-actor engagement framework outlined by Shawky, Kubacki, Dietrich and Weaven [10] linking online engagement to behavior. The current study’s focus on social media advertising effectiveness is important theoretically and practically. Theoretically, the current study extends social media evaluation frameworks linking online engagement metrics with behavioral actions. On a practical level, the study enables advertisers to create effective behavior change messages on social media that go beyond likes, comments and shares, delivering empirical evidence outlining the most effective approach to engage audiences and drive action.

## 2. Literature Review

Advertisements are designed with the ultimate goal of changing behavior [13]. Commercially, advertisers aim to increase sales by encouraging customers to purchase certain products or choose specific brands [8,14,15]. Beyond commercial application, the power of advertising is harnessed to positively change people’s lives, encouraging social and health behaviors such as quitting smoking [14], encouraging healthy eating [15], preventing diseases [16], safe driving [17] and increasing charity donations [18]. Such efforts are known as social advertising, the use of promotion and communication techniques to change social behavior [19]. To motivate the adoption of positive social behavior, social advertising raises awareness, induces action and reinforces maintenance of prosocial behaviors [19,20]. One area where social advertisers may deliver change is the impact of low-quality donations on charities and the environment. One of the most challenging tasks for charities is filtering donations received based on their quality before moving donated goods for redistribution or sale to generate revenue to support essential charity service provision [21]. Australian charity organizations spend millions of dollars each year on donation sorting processes, ensuring that unusable items donated to charities are discarded and others are remanufactured, while the remaining goods are distributed or sold. In 2018, over USD 9 million was spent sending unusable donations to landfill [22]. Processing of waste by charities diverts funding away from the delivery of essential community services [21]. As much as 30% of goods donated are estimated to be unusable, suggesting that there is substantial room for improvement. Despite the magnitude of the problem, limited research focused on improving the quality of donated items is available [23].

### 2.1. Emotional Advertising Appeals

As advertisers increasingly seek greater communication effectiveness, the choice of advertising appeal requires more consideration and careful assessment [24]. Viewers may utilize cognitive or affective evaluation systems when processing an advertisement message [25]. Rational appeals rely on cognitive evaluations through the persuasive power of arguments or reason to change audience beliefs, attitudes and actions. Such messages are evident in the dissemination of scientific information such as the ones seen during the COVID-19 pandemic. These include facts, infographics and arguments that appeal to a person’s rational processes [5]. Conversely, emotional appeals utilize the affective evaluation system by evoking emotions to drive action. Recent meta-analytic studies identified that consumers respond more favorably to emotional appeals compared to rational appeals [9,26].

Emotions are defined in many different ways in the literature. In an effort to summarize all definitions in one, Kleinginna and Kleinginna [27] provide a unified definition that is now relied on by psychology, marketing and other disciplines. They define emotions as “a complex set of interactions among subjective and objective factors, mediated by neural/hormonal systems, which can (a) give rise to affective feelings of arousal, pleasure/displeasure; (b) generate cognitive processes such as emotionally relevant perceptual effects, appraisals, labeling processes; (c) activate widespread physiological adjustments to the arousing conditions; and (d) lead to behavior that is often, but not always, expressive, goal-directed, and adaptive (p. 371)”. For decades, scholars have been studying the effect of emotion on behavior through multiple disciplines and contexts. In the past 10 years, considerable advancement has been clear in research around emotions. Specifically, technology advancements along with new research methodologies allowed scholars to measure and track emotion effects more accurately than before. For example, autonomic measures, including facial expression, heart rate and skin conductance, enabled researchers and practitioners to test emotional responses to certain stimuli [28]. This, along with digital and social media growth over recent decades, created an opportunity for advertisers to create, manipulate and test different advertising strategies to achieve the highest persuasion effects.

Researchers from psychology, social sciences, health and marketing agree on the crucial role emotions play in shaping human behavior [29,30,31]. Emotions have been part of persuasion models as early as the AIDA model, with desire indicating an emotional reaction following the cognition level of attention and interest [32]. More recently, emotions were found to dominate cognition in a persuasion process, occurring before any cognitive assessment of the message [33]. Hence, emotions are crucial in advertising’s ability to influence behavior [30,31].

For years, classifying emotions has been a research interest with multiple schools of thought. There are two main ways of classifying emotions, categorically (i.e., discrete emotions) or dimensionally. The discrete emotions approach posits that emotions are specific and defined. Different scholars present different sets of discrete emotions. For example, Ekman [34] presented six basic emotions, namely anger, disgust, fear, joy, sadness and surprise. Plutchik [35] argued there are eight basic emotions (fear, anger, sorrow, joy, disgust, acceptance, anticipation and surprise), with mixed emotions producing a secondary emotion (e.g., anger and disgust produce hostility). Models based on the discrete emotions approach appeared, mapping emotions on different dimensions of valence and arousal [36]. On the other hand, the dimensional theory of emotions classifies emotions based on three main dimensions: (a) valance, (b) arousal and (c) dominance. Hence, emotions can be positive or negative, highly aroused or calm and dominating or under control. Application of the dimensional theory is seen in testing different emotional appeals in advertising with positive emotional appeals and negative emotional appeals, and more recently a mixture of both valanced appeals (i.e., coactive appeals) [1,7,37]. The dimensional theory allows for valid comparison of different advertising strategies and appeals and has proven to be valid in multiple empirical results [38,39,40]. Hence, the current study employs the dimensional theory of emotions in classifying emotional appeals.

Based on the dimensional theory of emotions, people can perceive any emotional appeal stimulus as pleasant, unpleasant or a mixture of both (i.e., coactive state) [41]. Hence, emotional appeals are categorized as positive, negative and coactive based on the valance of employed emotions. Emotional appeals research employing the dimensional theory of emotions focuses on the effect of different valanced emotions on cognitive and behavioral actions [42]. While there is a strong connection between emotional appeals and behavior change [43], inconsistent results are evident in the literature when comparing positive, negative and coactive appeals (e.g., [7,8,17,44,45]).

### 2.2. Positive, Negative and Coactive Appeals

While positive emotional appeals were found to increase an individual’s tendency to take action and yield higher message liking [9,46], they are explored and utilized to a lesser extent when contrasted with negative emotional appeals [47,48,49]. When positive appeals are studied, humor appeals remain the focus, with less attention directed to the utilization of other positive emotional appeals which may deliver behavioral change [9]. A review of the literature indicates that positive appeals hold a persuasive advantage in both social and commercial behavior. Wang et al. [50] found positive admiration appeals to increase purchase intentions more than negative appeals. Similarly, Vaala et al. [51] support the use of positive empowering appeals when targeting health-related behavior. Positive appeals are especially effective when targeting males [52], however, studies of positive appeal effectiveness remain limited in number and in execution [47,48]. Some limitations in positive appeals are discussed in the literature. Segev and Fernandes [53] found positive appeals to be only effective when the behavior requires low effort. Hence, when environmental or climate change advertisements encourage green consumption, recycling or other complex behaviors, positive appeals might be less effective. Similarly, when positive appeals are used to evoke hope in audiences, hope for change is reported in the viewers instead of action taken towards change, indicating an emotion-focused coping function [54]. Hence, social advertisers remain reluctant to apply positive approaches. Fewer examples of positive appeals being applied to address social issues such as alcohol consumption [55], obesity [56], the environment [52] and safe driving behaviors [17] are evident.

Negative appeals, on the other hand, dominate research and practice, with over 70% of social advertisements employing negative appeals [48]. As the main driver of psychic discomfort, negative appeals are utilized to create emotional imbalance to stimulate behavior change [57]. According to this view, a message that is negatively framed when aiming to drive donations to charities is designed to make the individual feel uncomfortable as they are blamed for the poverty of certain groups (e.g., homeless children). To eliminate such feelings, a viewer is then more likely to contribute to the solution by donating to the charity [58]. While the use of negative appeals has been found to be effective in multiple contexts, such as healthy eating [56], moderate alcohol consumption [55] and safe driving [17], certain limitations apply. Negative appeals result in developing a coping mechanism such as ignoring the message (i.e., flight) or rejecting the message (i.e., fight), reducing message effectiveness [30,31,59]. Furthermore, negative appeals dominate social advertising efforts [47,48], resulting in desensitization to negative emotions, potentially causing such appeals to become less effective [57]. Finally, negative appeals can serve to reinforce stereotypes, further stigmatizing some people which can lead to reactance in some areas of the community [16].

Appeals utilizing both positive and negative emotions are labeled inconsistently in the literature. For example, Hong and Lee [60] and Taute et al. [61] employ the term mixed emotional appeals while others utilized the term coactive appeal [7,8,62,63,64]. The current study employs the term coactive appeals as coactivity is used to explain the mixed state of emotions and is applied more heavily in the marketing communication literature [8,62,64]. When comparing single appeals with coactive appeals that feature an emotional shift (e.g., from positive to negative), coactive appeals were found to be more effective [65,66]. Hence, coactive appeals have recently gained research attention, with advertising studies including such appeals in their evaluations [7]. Coactive emotional appeals seek to induce both positive and negative emotions simultaneously or as a flow from one appeal to the other [42,49]. For example, a coactive message can take the viewer on an emotional journey either from negative to positive or from positive to negative. The use of a negative to positive emotional flow or a threat–relief emotional message is hypothesized to result in a stronger persuasion outcome [49]. A recent study by Gebreselassie Andinet and Bougie [67] found the flow from negative to positive appeals produced more desirable results than negative or positive appeals alone. Similarly, Rossiter and Thornton [68] found fear–relief appeals to reduce young adults’ speed choice when driving. This is due to positive appeals’ ability to reduce different defensive reactions (e.g., fight, flight) that negative appeals generate. When a positive appeal is added to a negative appeal, post-exposure discomfort is reduced, resulting in the combination of appeals (i.e., coactive) being more effective in changing behavior [66,69,70]. Nonetheless, negative appeals remain highly featured in social advertising messages. This is attributed to the rich action tendency potential negative appeals hold [71], along with their ability to activate the brain more than other emotions [72]. Negative appeals have the ability to drive action without being liked first. This explains their dominance in social advertising messages and heavy focus in the literature. No known study has empirically tested and contrasted the effectiveness of a coactive appeal with positive and negative appeals directly on social media platforms. Previously studies compared the three appeals using self-report data collection measures, an approach that is limited by social desirability effects [73]. This study eliminates such limitations by utilizing social media advertising tools and measures where data are collected based on the viewer’s actual reactions on the platform (e.g., likes and clicks) rather than intentions to perform such reactions [1,74].

### 2.3. Theory

This study applies and builds on the multi-actor engagement framework proposed by Shawky, Kubacki, Dietrich and Weaven [10]. The framework provides an “integrated, dynamic and measurable framework for managing customer engagement on social media” enabling marketers to understand the different levels of engagement and measure the success of their campaigns [10]. As social media grows beyond simple dyadic exchanges between customers and companies, the multi-actor engagement framework operationalizes the different levels of engagement in a multi-actor ecosystem where customers, fans, organizations and stakeholders all contribute to levels of engagement with content. The different levels of engagement set by Shawky, Kubacki, Dietrich and Weaven [10] include connection, interaction, loyalty and advocacy. Connection is defined as a one-way communication where content is presented to customers without any action taken by the customer. When the stimulus attracts a customer’s attention, connection is achieved. In other words, Schivinski et al. [75] label this level as the consumption stage where social media users consume content but do not necessarily interact with it. At this level, customers passively consume the online content without taking any action yet [75,76]. Based on the Shawky, Kubacki, Dietrich and Weaven [10] framework, this level is measured by reach and impressions. Reach is defined as the number of unique users who viewed an advertisement, while impressions are recorded every time an ad is viewed, including multiple views by the same user [77]. The next level in the Shawky, Kubacki, Dietrich and Weaven [10] framework is interaction, and this level highlights the beginning of two-way communication between different actors, including customers, organizations and other customers. At this level, users engage with the advertisement by interactively contributing to the advertised message [75]. Social media interaction can be defined as “the number of participant interactions stratified by interaction type” [78]. Interaction types include likes, comments, clicks and overall engagement which are utilized to measure this level following the Shawky, Kubacki, Dietrich and Weaven [10] framework. The third level in the Shawky, Kubacki, Dietrich and Weaven [10] framework is loyalty, where interaction is repeated over time. A user is regarded as loyal if they are consistently seen interacting and contributing to an organization’s advertisements and content on social media. To encourage loyalty on social media, an organization’s content should aspire to complement the user’s image, as this will increase the chances of interactions over time and sharing with others [79]. This level is measured by multiple comments and messages in the Shawky, Kubacki, Dietrich and Weaven [10] framework. Finally, advocacy marks the fourth and highest level of engagement. Advocacy is recorded when customers spread an organization’s message by generating new content through their networks. Advocacy is where interaction is sustained, and support moves beyond the dyadic nature, reaching users’ own networks. This is when users share organizations’ content with their community circles through their own pages, profiles and networks [80]. Following the Shawky, Kubacki, Dietrich and Weaven [10] framework, this level is measured by shares, tagging of others on a post and word of mouth. The last two levels are parallel to Schivinski, Christodoulides and Dabrowski [75] creation level where users generate and create content. This is regarded as the highest level of online engagement as it motivates future interaction and involvement with the organization online and offline [76].

**Figure 1:**
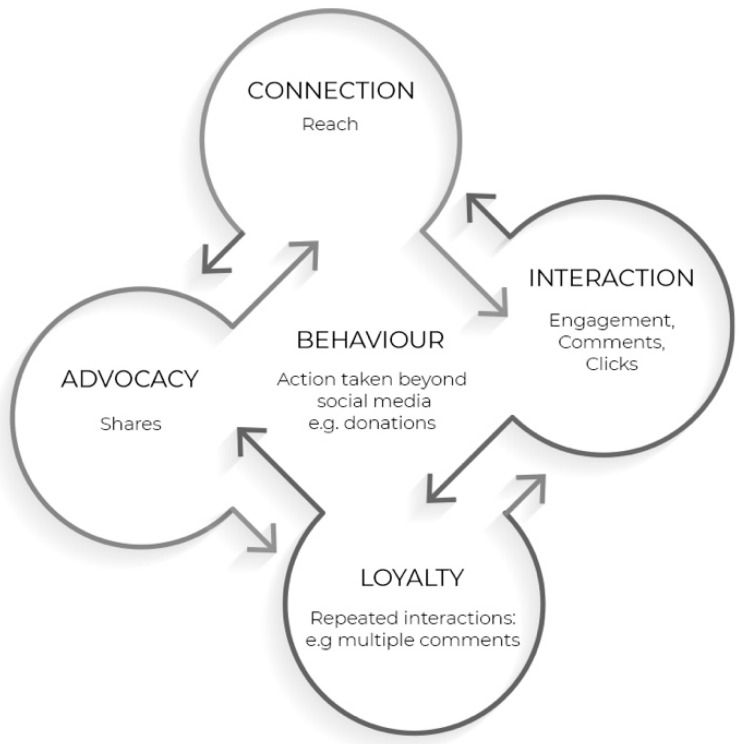
The extended multi-actor engagement framework [10].

The multi-actor engagement framework stops at advocacy as the highest level of engagement. As a result of our study, we propose a fifth level which marks the transition from online engagement to behavior. The fifth level signifies the customer’s action beyond social media platforms, and this can be reflected by purchases, donations, registrations, signatures on a petition and many other actions. While previous studies explore advertising effectiveness with customer perceptions such as attitudes, memorability, likability of the ad and intentions to take action, the direct effect of advertising on behavior, specifically in a social media context, is yet to be explored [71]. This is now possible with methodological advances and digital and social media platforms that allow for experiments to track not only automated measures on social media (e.g., likes and shares), but further action taken beyond such platforms (e.g., filling lead form, visiting a store, buying a product) [71]. This is of specific interest to advertisers employing emotional appeals, as each emotion has different action tendencies which influence the audience behavior after being exposed to the emotional advertisement appeal. As Poels and Dewitte [71] explain, different emotional appeals “help the individual sort out which action tendency is the most functional in this situation”. Each behavior is tracked differently, for example, weight loss campaigns can be tracked through the audience’s eating habits and exercise patterns, while antitobacco campaigns may track cigarette purchases, hence, this level has a number of possible measures. If measuring purchases of a product or donations to an online charity, the click through rate to the product or the donation page along with the number of orders or the amount of donations are examples of measures that reflect actions. For the purpose of this study, we measure actions through the number of requests to receive a donation sorting bag that helps reduce textile waste. The extended model is shown in Figure 1.

Past literature identified that positive advertising appeals produce an emotion-focused coping mechanism, while negative appeals create emotional imbalance to stimulate behavior change, and coactive appeals may reduce defensive reactions with less evidence of effectiveness in creating behavior change. For the purpose of the current study, we focus on four online behavioral outcomes. First, connection is defined by reach. Second, interaction is defined by engagement, likes, comments and clicks. Third, loyalty is defined by repeated actions on the ad. Finally, advocacy is defined by sharing the ad. Guided by past research, the current study expects negative advertising appeals to be more effective in evoking online behavioral responses than positive and coactive message on social media. Hence, the following hypotheses are proposed:

**Hypothesis** **1** **(H1):** *Negative advertising appeals will achieve more interactions than positive and coactive advertising appeals on social media*.

**Hypothesis** **2** **(H2):** *Negative advertising appeals will achieve more loyalty than positive and coactive advertising appeals on social media*.

**Hypothesis** **3** **(H3):** *Negative advertising appeals will achieve more advocacy than positive and coactive advertising appeals on social media*.

**Hypothesis** **4** **(H4):** *Negative advertising appeals will achieve more behavior actions than positive and coactive advertising appeals on social media*.

### 2.4. Gaps and Aims

This study aims to address three main gaps in the literature. First, the need for a social media advertisement evaluation model that addresses both online engagement as well as behavior actions which is addressed through an empirical study. Second, the evaluation of social media advertisements has been limited by self-reported measures of intentions to engage with advertisements (e.g., intention to click, like, share) [81], neglecting actual engagement measures (e.g., comments, reactions, shares, likes and ad clicks) that can be directly observed in social media. Third, current evidence on social media advertising appeals’ effectiveness in engagement and changing behavior remains conflicted, inconsistent and fragmented. Such gaps create a challenge for researchers aiming to understand social media advertising appeal effectiveness and advertisers, given that limited guidance is available to provide an implementation roadmap that can be relied upon to deliver behavior change benefitting people. This study addresses the aforementioned gaps testing the capacity of positive, negative and coactive advertising appeals to engage audiences on social media and drive behavioral actions.

## 3. Material and Methods

The current study employed an experimental study design, where three advertising appeals (positive, negative and coactive) were designed following Alhabash, McAlister, Hagerstrom, Quilliam, Rifon and Richards [7] and Hong and Lee [60] and published on Facebook following a pre-test conducted with a participant panel (*n* = 10).

### 3.1. Pre-Test

An online survey was distributed featuring the three advertisements. After exposure to each ad, participants were asked to rate how the advertisement made them feel on a 7-point scale (mostly positive/mostly negative) [82]. Next, participants were asked to describe how the advertisements made them feel in one word. The three ads maintained similarities in visuals and manipulated verbal elements to represent each appeal. The pre-test included one between-subjects ANOVA to compare advertisements’ emotional valance and a sentiment analysis of each advertisement response. The aim was to ensure that the positive advertisements were rated as more positive than the negative and coactive advertisements, the negative advertisements more negative than positive and coactive advertisements and the coactive advertisements in the middle. Moreover, the pre-test included a sentiment analysis of participants’ feedback on each advertisement. Word maps were generated and analyzed to confirm each ad represented the respective appeal.

### 3.2. Social Media Advertisements

**Figure 2:**
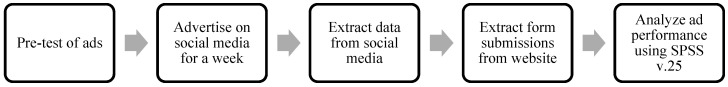
Research procedure.

After the pre-test, the three advertisements were published on Facebook and promoted for a week, controlling for the reach (i.e., number of people who were presented with the ad) of each advertisement through Facebook’s A/B testing tool on Facebook ads manager. The A/B testing tool allows for a comparable data set between the tested advertisements by controlling for reach across the different groups along with demographic elements (e.g., gender). Facebook ads manager allows for extraction of advertisements’ performance data as a spreadsheet, which was then used by the research team to analyze advertising appeal effectiveness using SPSS v.25. Facebook records advertisements’ performance data in key metrics including reach, likes, comments and shares. The ad appeared to Facebook users on their news feed as they scrolled through the content. The published ads (see Table 1) were linked to a charity website. One aim of the website is to educate people on what to donate to increase the quality of donations for Australian charities. When landing on the website, customers were asked to fill in a form to request a cloth sorting bag for their donations. Each form submission was recorded, and web data were extracted for all form submissions when the campaign was over. The research procedure is outlined in Figure 2.

### 3.3. Analysis and Measures

The three advertisements employed in this study were analyzed using Shawky, Kubacki, Dietrich and Weaven [10] multi-actor social media engagement framework. When customers viewed the advertisement, reach was recorded. When a customer liked or reacted to or clicked on an ad, interaction was recorded. When customers commented multiple times or replied to others to clarify the message or provide information, loyalty was recorded. When users shared the advertisement, advocacy was recorded. Finally, when customers filled in the form on the charity website, action was recorded. The form was created as a lead capture tool where customers filled in their information (e.g., name, address, contact details) to request a donation bag they could use to take their donations to charities. Three separate forms were created with three links for each advertising appeal. Data of each ad’s performance were extracted from Facebook ads manager while data of all request forms were extracted from the website after the ads on Facebook ended and were analyzed based on the number of requests received on each form. Following Merchant, Weibel, Patrick, Fowler, Norman, Gupta, Servetas, Calfas, Raste, Pina, Donohue, Griswold and Marshall [78], the data received were analyzed as categorical (out of all users reached, ad was liked: yes or no, ad was clicked on: yes or no) and continuous in the sense how many liked, clicked, commented. A chi-square test of independence was performed to examine the relation between advertising appeal and Shawky, Kubacki, Dietrich and Weaven [10] engagement levels: connection, interaction, loyalty, advocacy and the fifth proposed level of behavior, using SPSS v.25.

## 4. Results

**Figure 3:**
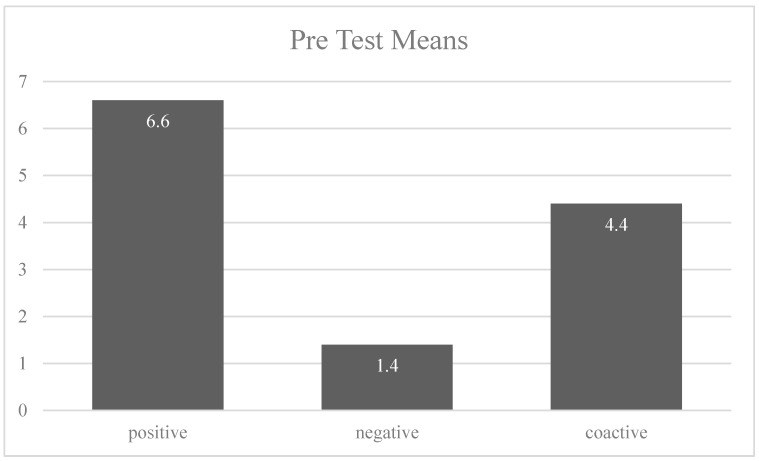
Pre-test mean scores of emotional valences.

A sample of ten participants was achieved for the pre-test with a mean age of 24 and balanced gender (50% females). Using SPSS v.25, pre-tests were successful for all advertising appeals. There was a statistically significant difference between group means showing a significant effect of appeal type on emotional valence (mostly positive/mostly negative) at the *p* < 0.05 level as determined by one-way ANOVA (F(2,27) = 199.957, *p* = 0.00). Post hoc analyses were conducted using Tukey’s post hoc test. The test showed that the three advertising appeal groups differed significantly at *p* < 0.05. The positive appeal ad (M = 6.60, SD = 0.52) was significantly more positive than the negative ad (M = 1.40, SD = 0.51) and the coactive ad (M = 4.40, SD = 0.69). Similarly, the negative ad (M = 1.40, SD = 0.51) was significantly more negative than the positive (M = 6.60, SD = 0.52) and coactive ads (M = 4.40, SD = 0.69). The coactive ad means appear in the middle as their mean scores were mostly neutral compared to the other two categories of emotional tone (see Figure 3). This result indicated that in the coactive condition, participants perceived the advertisement as both positive and negative at the same time, reflecting the bi-dimensional nature of the appeal (negative and positive).

**Figure 4:**
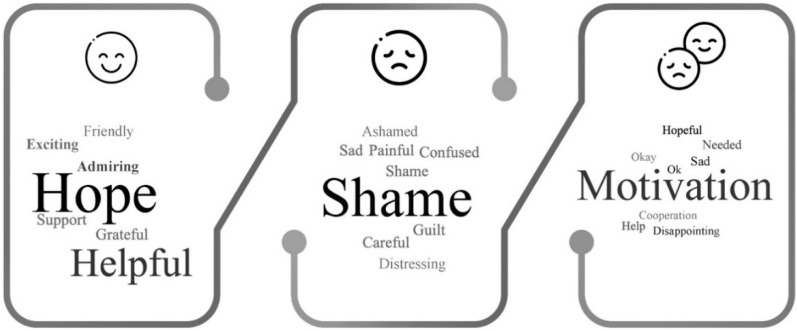
Word map based on sentiment analysis.

The sentiment analysis showed that the positive appeal was perceived as mostly hopeful, the negative mostly shameful and the coactive was perceived as motivational. Figure 4 showcases the word map for each advertisement.

### 4.1. Social Media Advertisements

**Figure 5:**
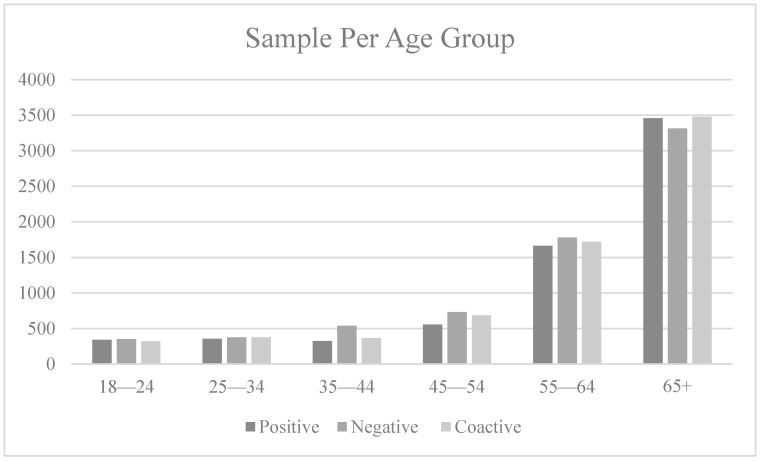
Gender distribution across the three ads.

**Figure 6:**
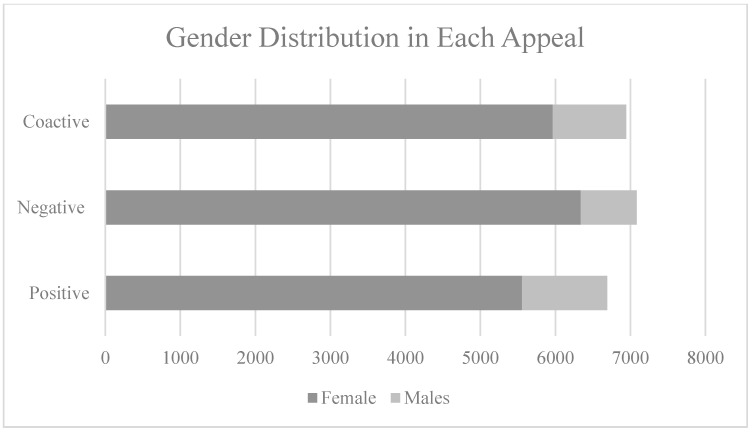
Gender distribution across advertisements.

**Figure 7:**
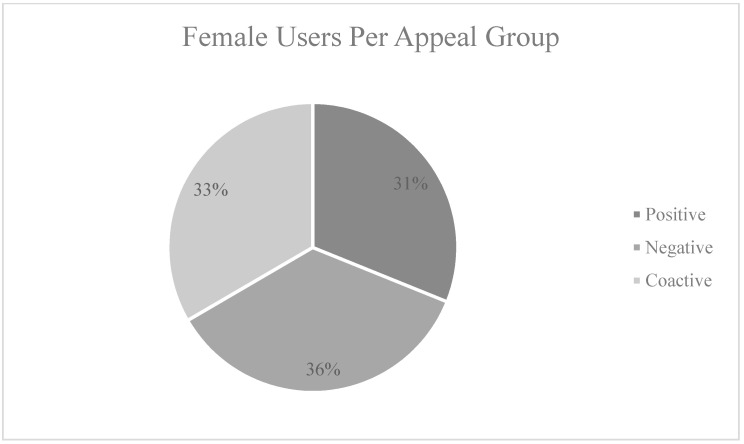
Distribution of females across the three appeals.

The three promoted appeals achieved a total of 23,905 impressions and reached 21,054 users which resulted in 787 clicks to the website. Facebook ads manager targeted a balanced sample for the three promoted appeals by using its A/B testing tool. The three ads reached Facebook users above 18 years of age of both genders (see Figure 5 and Figure 6). While the overall sample is female skewed (see Figure 6), each advertising appeal achieved a balanced reach for both genders (see Figure 7). The click through rate achieved through the three ads of 3.28% is considered above the average of 1.24% for Facebook ads [83]. A total of 28 requests were received for donation bags through the website forms. The results for each advertising appeal are discussed next.

#### 4.1.1. Connection

Connection was measured through reach and was controlled between the three appeal ads to ensure applicable comparison (see Table 2). A chi-square test of independence revealed an insignificant effect of appeal type on reach between the three advertisements χ^2^ (2, N = 21,054) = 4.57, *p* = 0.11.

#### 4.1.2. Interaction

Appeal type had a significant effect on the level of interaction. This is evident through all three measures: clicks, engagement and comments (see Table 3). The negative appeal ad had significantly more engagement than the positive and coactive appeals. A chi-square test of independence showed significance for clicks χ^2^ (2, N = 21,054) = 18.57 *p* < 0.05, and engagement χ^2^ (2, N = 21,054) = 20.68 *p* < 0.05. No significant difference was observed for comments χ^2^ (2, N = 21,054) = 4.94 *p* < 1, partially supporting H1. When comparing clicks on the positive and coactive appeals, no statistical significance was recorded at the 0.05 level χ^2^ (1, N = 13,851) = 1.49 *p* = 1.11. Similarly, effect was insignificant when comparing engagement χ^2^ (1, N = 13,851) = 1.48 *p* = 1.15 and comments χ^2^ (1, N = 13,851) = 1.02 *p* = 0.3 between positive and coactive appeals.

#### 4.1.3. Loyalty

No repeated interaction was recorded for any of the three advertising appeals (see Table 4). Therefore, appeal type had no effect on level of loyalty. Hence, H2 was not supported.

#### 4.1.4. Advocacy

A chi-square test of independence showed no significant effect of appeal type on advocacy (see Table 5). This is seen in the number of shares the three appeals received χ^2^ (2, N = 21,054) = 1.82 *p* = 0.40. Hence, H3 was not supported.

#### 4.1.5. Behavior

Behavior was measured through the number of requests for a donation sorting bag received for each advertising appeal. The negative appeal achieved the highest number of requests, followed by positive and coactive appeals (see Table 6). A chi-square test of independence showed a significant difference for appeal type based on the number of bag requests χ^2^ (2, N = 21,054) = 6.54 *p* < 0.05, supporting H4.

## 5. Discussion

The current study contributes to the literature in three ways. Firstly, we tested positive, negative and coactive appeals’ effectiveness on social media to understand their effect on engagement and behavior. This is the first study to directly examine advertising appeals on social media without the use of self-report measures. Our findings support the use of negative appeals over positive and coactive appeals when aiming to drive engagement and change behavior. This provides clear guidance for practitioners aiming to create effective social advertisement messages on social media. Secondly, this is the first study to apply and build on the Shawky, Kubacki, Dietrich and Weaven [10] social media multi-actor engagement framework in testing advertising appeals’ effectiveness. Our findings support the use of the framework in testing advertising effectiveness and show clear measures for each level of engagement. Finally, the study proposed an extension to the social media multi-actor engagement framework [10] with a clear and practical way of measuring actions beyond social media engagement. This will enable social advertisers to measure advertising effectiveness on actual behavior, moving beyond indirect behavioral measures such as attitudes, norms and intentions. Each contribution will be discussed in detail next.

### 5.1. Negativity Increases Appeals’ Effectiveness

**Figure 8:**
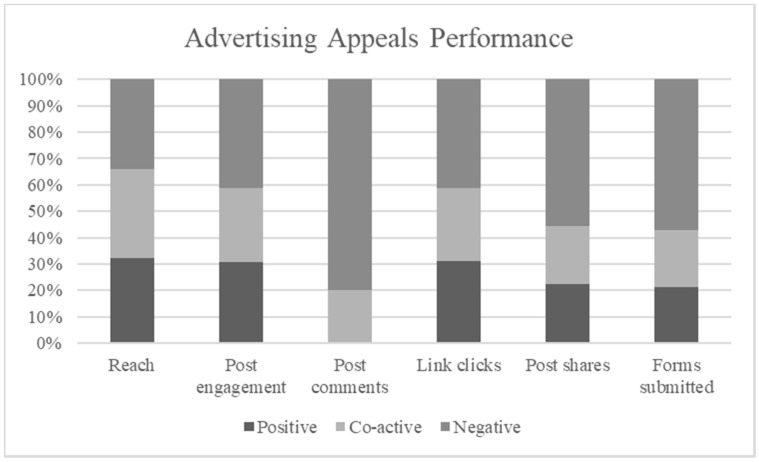
Comparison of advertising appeal performance.

When comparing positive, negative and coactive appeals’ performance in driving engagement and action on social media, our findings suggest negative appeals hold a persuasive advantage (see Figure 8). This is evident in the significant increase in engagement and actions for the negative advertisements when compared with the positive and coactive advertising appeals. This is consistent with previous findings, especially with behavior related to charities [18,84] and the environment [52,85]. Our findings support the limited effectiveness of positive appeals when complex issues are discussed. The advertisements employed by the current study address the issue of waste and its impact on the planet, where positive appeals have been found to be less effective in the past [54].

The effectiveness of coactive appeals has not been tested directly on social media before, marking a significant contribution of this study. Interestingly, the coactive appeal was equally effective when compared to positive appeals in attracting comments, and driving loyalty, advocacy and behavioral actions. The findings in this study indicate that both positive and coactive appeals performed in a similar way, contradicting previous findings supporting positive appeals’ effectiveness over coactive appeals [7]. The limited effectiveness of both positive and coactive appeals in this study may be attributed to the advertising platform.

### 5.2. Platform Effect on Appeal Effectiveness

Social media advertising engagement differs across social media platforms [86,87]. To understand why negative appeals were most effective in our experiment, a review of the platform of choice (i.e., Facebook) was necessary. Evidence suggests Facebook is among the most negative platforms in nature. Voorveld, van Noort, Muntinga and Bronner [86] explain the implications of such findings by relating them to advertising valence (i.e., appeals). Hence, when advertising on Facebook, advertisements that evoke negative feelings (i.e., negative appeals) perform best. The effectiveness of such appeals stems from the fluency between advertising appeal and platform nature [87]. Platform–appeal fit was found to increase the effectiveness of advertising and drive higher rates of engagement [87]. In fact, Facebook ran an experiment in 2014, where content was manipulated for half a million users. The experiment tested whether what users share is affected by what they see in their newsfeed. The findings supported the concept of platform–appeal fit. When negative content was increased in users’ feeds, their posts became more negative as a result [88]. Recently, Facebook was criticized for carrying out such experiments, which can have an impact on mental health, personal decisions and in many cases political election outcomes [89]. All of this results in users’ skepticism of the platform, contributing to its negative nature.

### 5.3. Driving Action Beyond Social Media Engagement

**Figure 9:**
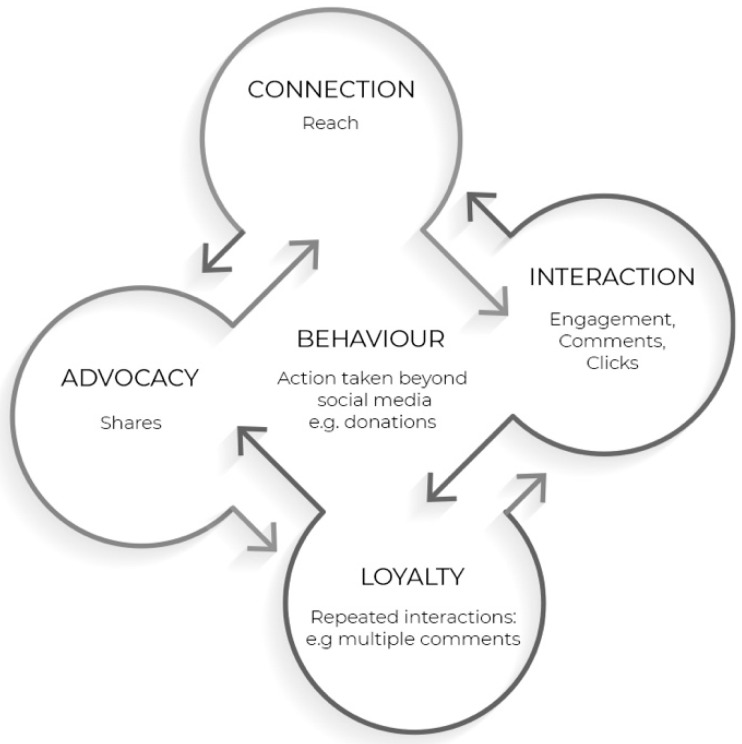
Proposed extension to Shawky et al. (2020) social media multi-actor engagement framework.

The current study applies and builds on the Shawky, Kubacki, Dietrich and Weaven [10] social media multi- actor engagement framework. The framework provides a practical tool for organizations seeking to evaluate their social media content. This study applies the framework, testing advertising appeals’ effectiveness, an application of the framework that has not previously occurred. It showcases the ability to use the Shawky, Kubacki, Dietrich and Weaven [10] framework as a tool to evaluate social media advertising effectiveness in driving engagement and prosocial behaviors. While the framework proved to be a practical and easy measurement tool for advertisers, it has a key limitation when aiming to carry out a thorough evaluation of advertising messages’ capacity to drive action. Linking online engagement to behavior remains limited when evaluating social media advertising effectiveness. Hence, an extension to the model is proposed, where behavior is measured through action (e.g., donations), extending understanding beyond social media engagement (see Figure 9). This extension could be tested empirically, allowing actions taken to be observed and analyzed. Behavior could be measured as a fifth step in Shawky et al.’s framework.

Negative appeals achieved the highest actions, while positive and coactive appeals received equal actions from users. This could be explained by the emotion-focused coping function, where positive emotions produce a hope for change rather than inducing a motivation to take action [54]. Furthermore, when viewers hold favorable prior attitudes towards the advertised behavior (i.e., reduce waste) positive appeals are found to be less effective [90]. While there are no data on prior attitudes of the viewers for this experiment, the comments received about the negative appeal advertisement suggest some people are passionate about the environment, and they want to take actions to help others and reduce waste. Hence, negative appeals were more effective in driving both engagement and action.

### 5.4. Limitations and Future Research

Three main limitations apply to this study. First, mediators of effectiveness, such as prior attitude towards the issue, were not examined in this study. Future research is recommended to employ a pre-exposure survey to collect such data or utilize social media targeting tools to target specific audiences with certain interests. Second, the advertisements tested in this study were shared predominantly on Facebook where negative content dominates. Future research should investigate other platforms to understand the effects that social media platforms exert on advertising appeal effectiveness. Empirical tests of different appeals on multiple platforms (e.g., Twitter, TikTok, Snapchat) are needed to draw conclusions on where different appeals perform best. Third, the sample reached by this study may be small when compared to other studies in social media settings [91], and future research may increase the reach by increasing the budget invested in the advertising campaign on Facebook.

It is important to note that different contexts may achieve different results, and the experiment can be replicated to understand if other behaviors, such as the uptake of the COVID-19 vaccine, are more effectively achieved through negative advertising appeals. Research shows a promising effect of emotional appeals in both social and health domains, with more empirical evidence needed [92]. Future research may investigate the role of social media advertisements in inspiring loyalty and advocacy through different emotional appeals. The current study found no effect of appeal type on loyalty and advocacy, presenting a limitation to the overall findings. To increase analytic rigor, future research may employ a CB/PLS-SEM approach to test social media advertising’s effect on behavior [93].

## 6. Conclusions

This study examined positive, negative and coactive advertising appeals’ effectiveness in driving engagement and actions on and beyond social media platforms. Findings support the use of negative advertising appeals over positive and coactive appeals. The results highlight how negative appeals on social media advertising in an environmental and charity context can deliver superior outcomes to engage more people and positively impact social behavior. Theoretically, this study highlights the value of using Shawky et al.’s (2020) multi-actor social media engagement framework to test social advertising appeal effectiveness and provides a practical extension to evaluate behavioral outcomes.

## Figures and Tables

**Table 1:** Overview of employed stimulus.

Appeal	Artwork
Positive	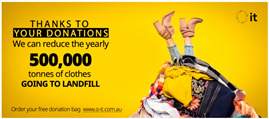
Negative	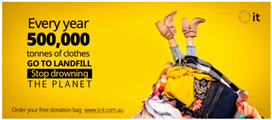
Coactive	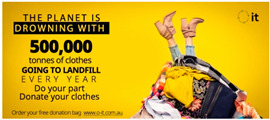

**Table 2:** Number of people reached compared to impressions on positive, negative and coactive appeals.

	Reached: Yes	Reached: No	Total
Positive	6773	1030	7803
Negative	7203	1021	8224
Coactive	7078	800	7878

**Table 3:** Number of interactions on positive, negative and coactive appeals.

	Clicked: Yes	Clicked: No	Total (Reach)
Positive	244.00	6529	6773
Negative	323.00	6880	7203
Coactive	220.00	6858	7078
	**Engaged: Yes**	**Engaged: No**	**Total (Reach)**
Positive	261	6512	6773
Negative	352	6851	7203
Coactive	240	6838	7078
	**Commented: Yes**	**Commented: No**	**Total (Reach)**
Positive	0	6773	6773
Negative	4	7199	7203
Coactive	1	7077	7078

**Table 4:** Number of repeated interactions on positive, negative and coactive appeals.

	Repeated Action: Yes	Repeated Action: No	Total (Reach)
Positive	0	6773	6773
Negative	0	7203	7203
Coactive	0	7078	7078

**Table 5:** Number of shares on positive, negative and coactive appeals.

	Shared the Ad: Yes	Shared the Ad: No	Total (Reach)
Positive	2	6771	6773
Negative	5	7198	7203
Coactive	2	7076	7078

**Table 6:** Number of requests on positive, negative and coactive appeals.

	Form Submitted: Yes	Form Submitted: No	Total
Positive	6	6767	6773
Negative	16	7187	7203
Coactive	6	7072	7078

## Data Availability

Data available upon request.
